# Has noninvasive prenatal testing impacted termination of pregnancy and live birth rates of infants with Down syndrome?

**DOI:** 10.1002/pd.5182

**Published:** 2017-12-29

**Authors:** Melissa Hill, Angela Barrett, Mahesh Choolani, Celine Lewis, Jane Fisher, Lyn S. Chitty

**Affiliations:** ^1^ Genetics and Genomic Medicine UCL Great Ormond Street Institute of Child Health London UK; ^2^ North East Thames Regional Genetics Service Great Ormond Street Hospital for Children NHS Foundation Trust London UK; ^3^ Department of Obstetrics and Gynaecology, Yong Loo Lin School of Medicine National University of Singapore Singapore; ^4^ Antenatal Results and Choices (ARC) London UK

## Abstract

**Background:**

Implementation of noninvasive prenatal testing (NIPT) as a highly accurate aneuploidy screening test has raised questions around whether the high uptake may result in more terminations of pregnancies and fewer births of children with Down syndrome (DS).

**Aim:**

The aim of the study was to investigate the impact of NIPT on termination and live birth rates for DS.

**Methods:**

Literature reporting pregnancy outcomes following NIPT was reviewed. Termination rates were calculated for women with a high‐risk NIPT result for DS. Two audits of pregnancy outcomes where NIPT indicated DS were conducted in the United Kingdom and Singapore.

**Results:**

Fourteen studies from the United States, Asia, Europe, and the United Kingdom were included in the review. Live births of children with DS were reported in 8 studies. Termination rates following NIPT were unchanged or decreased when compared to termination rates prior to the introduction of NIPT. Audits found 15 of 43 women in the United Kingdom and 2 of 6 in Singapore continued pregnancies following a high‐risk NIPT result.

**Conclusions:**

Termination rates following the detection of DS by NIPT are unchanged or decreased compared to historical termination rates. Impact on live birth rates may be minimal in settings where termination rates fall. Population‐based studies are required to determine the true impact.

## Abstract 2

**WHAT IS ALREADY KNOWN ABOUT THIS TOPIC?**

- Noninvasive prenatal testing (NIPT) has been shown to be a highly accurate prenatal screening test for DS and is being implemented widely throughout the world.
- Introduction of NIPT has increased the prenatal detection of DS with a significantly reduced invasive testing rate, but the impact on rates of termination of pregnancy and the number of children born with DS is not yet known.

**WHAT DOES THIS STUDY ADD?**

- Introduction of NIPT has a variable effect on termination rates for DS, but rates have remained unchanged or decreased when compared to termination rates reported prior to the introduction of NIPT, with many parents using NIPT for information and continuing pregnancies when results show a high risk of DS.
- Practical and emotional support structures are needed for these families.
- Where termination rates fall NIPT may have a minimal impact on live birth rates for DS.
- Monitoring at population levels is required for a more accurate assessment of live birth rates.

## 1 INTRODUCTION

Noninvasive prenatal testing (NIPT) for aneuploidy based on analysis of cell‐free DNA (cfDNA) in the maternal plasma became available in the private sector in 2011 and is now being offered widely throughout the world.^1^ NIPT is a highly accurate screening test that can be used from 10 weeks in pregnancy to detect Down syndrome (DS) (Trisomy 21) with high sensitivity (99%) and specificity (99.5%).^2^ However, NIPT is not diagnostic, and confirmation of a positive result by invasive testing (chorionic villus sampling or amniocentesis) is recommended.^3^ NIPT has been shown to be accurate in both the high‐risk and general pregnancy populations,^4^ and the use of NIPT as a screening test has been endorsed by professional bodies from several countries who encourage provision of pretest and posttest counseling to support informed choice.^3^, ^5^ NIPT has a much greater sensitivity than traditional screening methods such as the combined test that measures nuchal translucency and maternal serum biochemistry and a growing number of studies have confirmed that the introduction of NIPT into the screening pathway has significantly reduced the need for invasive testing.^6^, ^7^, ^8^, ^9^, ^10^

Overall, key stakeholders, such as pregnant women and health professionals, are very positive about the introduction of NIPT and highlight benefits such as safety, accuracy, and the detection of pregnancies affected with DS that would have been missed with traditional screening.^11^, ^12^, ^13^, ^14^ One of the questions raised in the literature about the widespread implementation of NIPT for DS is whether it will lead to an increase in the number of parents seeking prenatal testing and termination of pregnancy with a resultant significant decrease in the number of children born with DS.^11^, ^12^, ^15^, ^16^, ^17^ Research with pregnant women^11^, ^12^ and parents of children with DS^16^, ^17^ identified concerns that fewer children being born with DS could result in a reduction of social supports and resources for children with disabilities and a society that is less tolerant of people who have children with disabilities. Furthermore, these key stakeholder groups felt that a less tolerant society could increase feelings of pressure for pregnant women to have testing and subsequently terminate an affected pregnancy.^11^, ^12^, ^16^

Interest in and uptake of NIPT is high,^7^ making it likely that the number of parents opting for prenatal testing for DS will grow as many parents who would not have previously opted for prenatal testing because of the risk of miscarriage would be willing to have NIPT.^11^, ^18^ It is not yet clear, however, whether the increase in the numbers of women having prenatal testing will directly result in more terminations of pregnancy. There will be wide variation in the number of children born with DS between and even within countries depending on attitudes to prenatal testing, disability, and termination that are influenced by religious, social, and cultural settings, costs of prenatal testing and access to termination of pregnancy. Ultimately, we will see the impact of NIPT on the number of children born with DS from long‐term population‐based studies comparing live birth rates before and after the introduction of NIPT. These population‐based studies are crucial as they reflect the number of children born to parents who have chosen to continue the pregnancy after a prenatal diagnosis of DS as well as the number of children born to parents who decided not to have prenatal diagnosis and then had a baby with DS.

Several studies conducted prior to the introduction of NIPT have reported the number of children born with DS compared to expected numbers based on the prevalence of DS in the general population. These figures vary between countries: in the United States there were 30% fewer individuals with DS (2007),^19^ 50% fewer in the Netherlands (2015),^20^ 48% fewer in England and Wales (2008),^21^ 55% fewer in Australia (2004),^22^ 94% fewer in Taiwan (2010),^23^ and 55% fewer in China (2011).^24^ As the numbers of older women giving birth have grown, the numbers of pregnancies affected with DS have also increased. However, in England and Wales^21^ and in Europe^25^ the live birth rate remained relatively unchanged between 1990 and 2009 even though prenatal screening and diagnosis became more common over the same period. In contrast, in the United States, the live birth rate has increased since the early 1990s through to 2007.^19^ One small regional study conducted in the Hampton Roads area of Virginia reported that NIPT has not affected the number of children born with DS in this area.^7^ More time is needed to see the impact of NIPT on the live birth rate more widely and conclusively.

While we await definitive population‐based studies, there are several lines of evidence available to us now that can help assess the impact the introduction of NIPT might ultimately have on the numbers of parents choosing to continue their pregnancy following a prenatal diagnosis of DS. Here we examine the literature to look at reports of pregnancy outcomes following NIPT. We also describe 2 new audits of NIPT services in clinical practice that were conducted in England and in Singapore.

**What is already known about this topic?**

- Noninvasive prenatal testing (NIPT) has been shown to be a highly accurate prenatal screening test for DS and is being implemented widely throughout the world.
- Introduction of NIPT has increased the prenatal detection of DS with a significantly reduced invasive testing rate, but the impact on rates of termination of pregnancy and the number of children born with DS is not yet known.

**What does this study add?**

- Introduction of NIPT has a variable effect on termination rates for DS, but rates have remained unchanged or decreased when compared to termination rates reported prior to the introduction of NIPT, with many parents using NIPT for information and continuing pregnancies when results show a high risk of DS.
- Practical and emotional support structures are needed for these families.
- Where termination rates fall NIPT may have a minimal impact on live birth rates for DS.
- Monitoring at population levels is required for a more accurate assessment of live birth rates.

## 2 METHODS

### 2.1 Literature review

We reviewed the published literature reporting the number of live births of children with DS following screening with NIPT. A search of English‐language articles from the time NIPT entered clinical practice (January 1, 2011, to September 25, 2017) was conducted. We searched the PubMed electronic database using the following search terms: “cell free fetal DNA,” “NIPT” or “Non‐invasive prenatal test*” or “noninvasive prenatal test*” or “NIPD” or “non‐invasive prenatal diagnosis” or “noninvasive prenatal diagnosis.” A manual search of the reference lists of included studies and relevant original and review articles was also performed. Publications were included if they described data on numbers of women having NIPT for DS and provided information on pregnancy outcomes, such as live births, pregnancy termination, fetal demise, or stillbirths. Studies that described modelled data were excluded. The search identified 1726 articles. Titles and abstracts were examined by 1 reviewer (M.H.), and full‐text articles were obtained for 87 potentially relevant articles. Sixteen articles describing 14 studies met the inclusion criteria.^8^, ^26^, ^27^, ^28^, ^29^, ^30^, ^31^, ^32^, ^33^, ^34^, ^35^, ^36^, ^37^, ^38^, ^39^, ^40^ Formal quality appraisal of individual studies was not undertaken as the data sought generally comprised only a small component of the overall study. Data describing the study setting and the pregnancy outcomes were independently extracted by 2 reviewers (M.H., A.B.). A narrative synthesis of studies was then performed. Termination rates were calculated as a proportion of all pregnancies with a high‐risk NIPT result (excluding false positives and negatives). This includes cases confirmed with invasive testing and those confirmed at birth without confirmatory invasive testing. Where additional data were available, termination rates were calculated for women going directly to invasive testing.

A separate search was conducted to identify published termination of pregnancy rates prior to introduction of NIPT. Termination rates were sought for each of the countries where the studies included in the review were conducted. The PubMed electronic database was searched using the following terms: “*country name*” AND “Down* syndrome” AND “termination” or “abortion” or “live‐birth rates.”

### 2.2 Audit of pregnancy outcomes following NIPT as a clinical service in Singapore

NIPT has been offered at the National University Hospital Singapore since April 2014. NIPT is offered to all patients as an out‐of‐pocket test, with no subsidies. Invasive testing is recommended for confirmation of a high‐risk NIPT result. Other criteria for offering invasive diagnostic testing include a previous affected pregnancy, a high‐risk combined screening test, advanced maternal age, and structural abnormalities. A retrospective audit of pregnancy outcomes for women who chose to have NIPT or went straight to invasive testing without prior NIPT from April 1, 2014, to January 31, 2017, was conducted (DSRB number 2016/00253). Termination rates were calculated as a proportion of all pregnancies that had a high‐risk NIPT result (false positives and negatives excluded). Termination rates were also calculated for women going directly to invasive testing without NIPT.

### 2.3 Audit of pregnancy outcomes following NIPT offered as a clinical service in the United Kingdom

Following the development and validation of a cfDNA sequencing protocol by our NHS service laboratory (North East Thames Regional Genetics Service), NIPT was initially offered as part of a research trial in 8 UK maternity units, which demonstrated that NIPT could be successfully offered as a contingent test without increasing costs in the NHS.^35^ One weakness of this study was that there was potentially more pretest counseling offered by the research team than might be available in clinical practice. To evaluate informed choice in routine maternity care the study was extended and NIPT was offered by local maternity staff trained to discuss NIPT in North Thames units to women with combined test risk of ≥1/150 from March 1, 2015, to October 31, 2016. Invasive testing to confirm a high‐risk NIPT result was recommended. An audit of pregnancy outcomes was conducted (registered at Great Ormond Street Hospital NHS Foundation Trust as a service evaluation). The laboratory database of NIPT tests performed was reviewed, and a request was made to each referring hospital to give details of the pregnancy outcomes of all women who chose to have NIPT. Termination rates were calculated as a proportion of all pregnancies with a high‐risk NIPT result (excluding false positives and negatives).

## 3 RESULTS

### 3.1 Literature review of pregnancy outcomes following NIPT

Fourteen studies were included in the review, 8 were prospective^8^, ^30^, ^31^, ^32^, ^34^, ^35^, ^36^, ^37^, ^39^ and 6 were retrospective audits.^26^, ^27^, ^28^, ^29^, ^33^, ^38^, ^40^ Most studies were conducted in the United States (n = 5),^8^, ^28^, ^29^, ^31^, ^33^ however, 2 studies from the United Kingdom^35^, ^36^, ^37^ and China,^32^, ^40^ and individual studies from Hong Kong,^26^, ^27^ Taiwan,^30^ the Netherlands,^34^ Spain,^38^ and France^39^ were also identified. The 14 studies were diverse in their objectives and study design. Taking a broad overview of objectives, 4 studies reviewed the experience of offering NIPT at a single centre, looking at factors such as patient characteristics and uptake of NIPT and invasive testing,^28^, ^29^, ^31^, ^38^ one study was questionnaire based across 4 centres and explored women's views about NIPT and factors influencing decision making, including views on termination of pregnancy.^8^ One study looked at the utility of using NIPT for twin pregnancies.^39^ Four studies aimed to examine NIPT performance,^26^, ^27^, ^30^, ^32^, ^40^ and 3 explored the impact of implementing NIPT as part of state supported health care services.^34^, ^35^, ^36^, ^37^ Only 1 study had the explicit aim of looking at clinical outcomes and patient choices following NIPT, including continuing an affected pregnancy or opting for termination of pregnancy.^33^

Pregnancy outcome data from each of the studies are summarised in Table 1. A significant proportion of pregnancies where NIPT indicated a high risk of DS resulted in live births of infants with DS in each of the studies from the United States, the United Kingdom, and the Netherlands; however, there were no live births in the studies from China, Hong Kong, Taiwan, France and Spain. It is important, however, to consider that the numbers reported in some studies were very small. Notably, in 7 studies^8^, ^29^, ^31^, ^33^, ^35^, ^37^, ^40^ there were women with NIPT results indicating that the baby had a high risk of having DS who declined the offer of invasive testing to confirm the NIPT result.

**Table 1:** Summary of studies reporting outcomes of pregnancies following NIPT

Citation	Country	Study Design	NIPT Offered To	NIPT Tests Performed	Pregnancies with NIPT Suggesting DS	NIPT Confirmed by Invasive Testing	Termination	Miscarriage/Fetal Demise	Live Birth of Children with DS	Termination rate^a^
Lau et al^26^, ^27^	Hong Kong	Retrospective audit at 1 centre (Aug 2011‐Feb 2013)	All	1982	23	23	23	0	0	100%
Pettit et al^28^	United States	Retrospective audit at 1 centre (May‐Dec 2012)	High risk^b^	206	8	Not reported	5	0	3	63%
Vahanian et al^29^	United States	Retrospective audit at 1 centre (Mar‐Jul 2012)	High risk^b^	93	2	1	0	0	2	0%
Shaw et al^30^	Taiwan	Prospective study at 11 centres (June‐Dec 2012)	Very high‐risk (>1:30 or NT >3) or low‐risk (<1:1500) women having IPD	201	11	11	11	0	0	100%
Beamon et al^31^	United States	Prospective study at 1 centre (Jan‐Sept 2012)	High risk^b^	208	5	4	3	0	2	60%
Tiller et al^8^	United States	Prospective study at 4 centres (Mar‐May 2013)	High risk^b^	200	5	3	2	1	2	40%
Song et al^32^	China	Prospective study at 1 centre (May 2012‐Aug 2013)	High risk^b^	212	3	2	2	1	0	67%
Dobson et al^33^	United States	Retrospective audit at 2 centres (Mar 2012‐Dec 2014)	High risk^b^	Not reported (105 singletons/9 twins included in audit)	Singletons: 53 Twins: 5	Singletons: 38 (includes 3 false positive) Twins: not reported	Singletons: 32 Twins: 2	Singletons: 3 Twins: 2	Singletons: 14 (1 lost to follow‐up) Twins: 1	Singletons: 64% Twins: 40%
Oepkes et al^34^	The Netherlands	Prospective study at 21 centres (April‐September 2014)	High risk (≥1:200)	1211	31	31 (includes 2 false positive)	25	2	2	86%
Chitty et al^35^	United Kingdom	Prospective study at 8 centres (Nov 2013‐Feb 2015)	High risk (≥1:150) offered NIPT or IPD/intermediate risk (1:101‐1:2500) offered NIPT	2494	NIPT: 44 IPD: 29	36 (includes 1 false positive)	NIPT: 30 IPD: 27	NIPT: 3 IPD: 0	NIPT: 10 IPD: 2	NIPT: 70% IPD: 93% Overall: 79%
Gil et al^36^, ^37^	United Kingdom	Prospective study at 2 centres (Oct 2013‐Feb 2015)	High risk (≥1:100) offered NIPT or IPD/intermediate risk (1:101‐1:2500) offered NIPT	3698	NIPT: 16 IPD: 27	12	NIPT: 7 IPD: 25	NIPT: 0 IPD: 0	NIPT: 9 IPD: 2	NIPT: 44% IPD: 93% Overall: 73%
Gil et al^38^	Spain	Retrospective audit at 1 centre (Jan 2015‐Jan 2016)	High risk (1 > 250)	54	NIPT: 1 IPD: 4	1	NIPT: 1 IPD: 4	NIPT: 0 IPD: 0	NIPT: 0 IPD: 0	NIPT: 100% IPD: 100% Overall: 100%
Qiang et al^40^	China	Retrospective audit at 1 centre (Mar 2012‐Mar 2015)	High risk^c^	1901	15	14	13	1	0 (1 lost to follow‐up^d^)	87%
Le Conte et al^39^	France	Prospective study of tests performed at 1 laboratory (Nov 2013‐Aug 2015)	Twin pregnancies without ultrasound anomalies	492	4	4 (includes 1 false positive)	3	0	0	100%

a Termination rates were calculated as a proportion of all pregnancies that had a high‐risk result for DS from NIPT (false positives and negatives excluded). This may include cases confirmed with IPD and those confirmed at birth who did not undergo confirmatory IPD. Where additional data were available, termination rates were calculated for “NIPT”—those undergoing NIPT (including cases confirmed with IPD and those confirmed at birth without confirmatory IPD), “IPD”—those going directly to IPD without NIPT, and “Overall”—the termination rate for all cases of DS diagnosed prenatally.

b Definition of “high risk” includes; advanced maternal age, fetal ultrasound finding suggestive of aneuploidy, family history of aneuploidy, or positive maternal serum screening result. Aligned with American College of Obstetricians and Gynecologist (ACOG) Committee Opinion.

c NIPT was offered to women with a risk of one or more anomaly. All women had DS screening prior to NIPT.

d The case lost to follow‐up declined confirmatory invasive testing.

Abbreviations: DS, Down syndrome; IPD, invasive prenatal diagnosis; NIPT, noninvasive prenatal testing.

### 3.2 Audit of pregnancy outcomes following NIPT as a clinical service in Singapore

**Figure 1:**
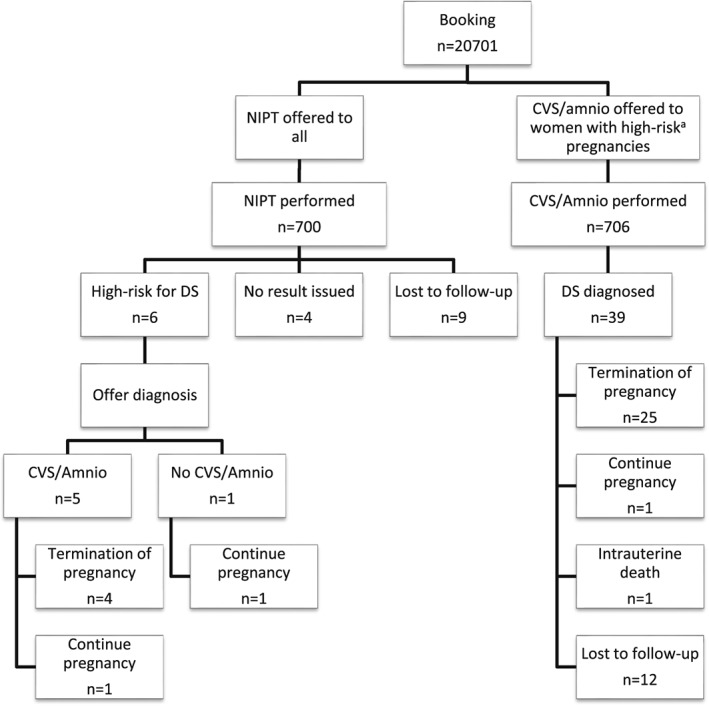
Flowchart showing numbers of women and outcomes for the Singapore audit

Between April 1, 2014, and January 31, 2017, there were 20 701 new bookings at National University Hospital Singapore; 700 women had NIPT (3.4%). For 6 women NIPT indicated that the baby had a high risk of being affected with DS. Five chose to confirm their results by amniocentesis, and the sixth declined further testing. There were no false positives. Of the 5 women with a confirmed pregnancy affected with DS, 4 opted for a termination (80%), and 1 continued the pregnancy. The woman who declined further testing following NIPT also continued the pregnancy. The termination rate for these women with high‐risk NIPT results for DS was 67%. Nine women were lost to follow‐up, and it was not possible to issue a result in 4 cases, because of high variance or low fetal fraction. Over the same period, 706 women (3.4%) opted for an invasive test without prior NIPT, and 39 had a positive result for DS. Twelve cases were lost for follow‐up; of the remaining, 27 women, 25 women chose to terminate their affected pregnancy (92.6%), including 1 woman who had a selective feticide for a single affected twin. There was 1 intrauterine death, and 1 continued pregnancy. Overall, from the 33 cases with confirmed DS following invasive testing or high risk NIPT where outcomes were known, 29 terminated (87.9%) and 4 (12.1%) continued with 1 being stillborn. A summary of the audit findings is presented in Figure 1.

### 3.3 Audit of pregnancy outcomes following NIPT offered as a clinical service in the United Kingdom

**Figure 2:**
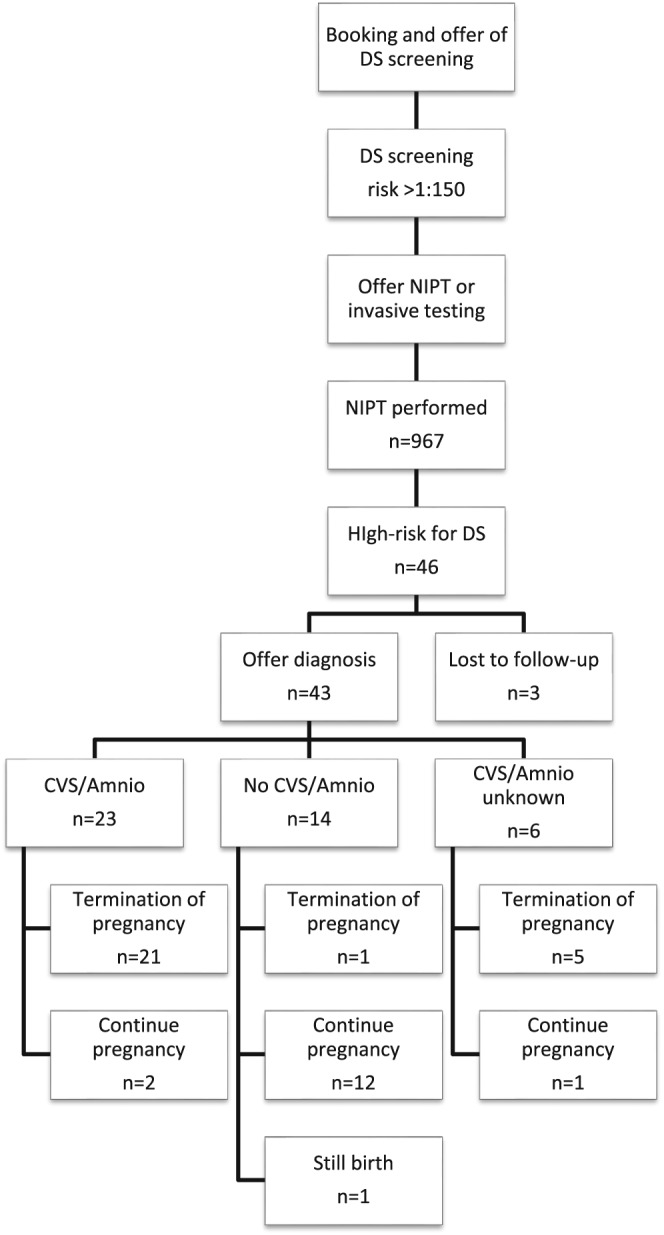
Flowchart showing numbers of women and outcomes for the UK audit

Nine hundred and sixty seven women with a high‐risk DS screening result in the North Thames Region had NIPT and 46 were found to be highly likely to have DS. There were no false positive DS results; however, 3 women in this group were lost to follow‐up. Of the 46 women found to be highly likely to have DS by NIPT, outcomes were available for 43. There were 27 (62.7%) women who chose to terminate the pregnancy, 1 had a still birth (2.3%), and 15 (34.9%) continued the pregnancy. Out of 15 parents who chose to continue the pregnancy, 12 did not have their NIPT result confirmed by invasive testing, 2 had invasive testing, and 1 decision was unknown. Twenty one of the 27 parents who chose to have a termination of pregnancy had invasive testing to confirm the NIPT result, 1 declined and the decision was not reported for 5. A summary of the audit findings is presented in Figure 2.

## 4 DISCUSSION

Our literature review and audits show that many women opting for NIPT who had a result indicating they were highly likely to have a baby with DS chose to continue their pregnancy. Although numbers are relatively small, when we look at the data as a whole our findings suggest that the high uptake of NIPT worldwide includes many women who would like additional information about their baby that will not necessarily be used for decision making about termination of pregnancy. In addition, several studies in the review reported that some women declined an invasive test to confirm the NIPT result and continued their pregnancy, further emphasising that many women choosing to have NIPT want information about the health of their baby, but would not risk miscarriage with an invasive test. Differences in the number of live births were seen between countries as women chose to continue pregnancies highly likely to be affected with DS in the United States, the United Kingdom, the Netherlands, and Singapore, but there were no confirmed live births reported in the studies from China, Hong Kong, Taiwan, France, and Spain.

Termination rates for DS prior to the introduction of NIPT vary quite markedly throughout the world. In the United States, a systematic review of termination rates in studies published between 1995 and 2011 found that 67% of women opted for termination of pregnancy following prenatal diagnosis of DS.^41^ Termination rates of over 90% have been reported in the United Kingdom,^21^ Australia,^42^, ^43^ China,^24^ and the Netherlands.^44^ In Taiwan, a termination rate of 67.5% was reported for 2001^45^; however, prenatal screening and diagnosis has since been more widely introduced and the live birth rate for children with DS decreased from 48.7% in 2001 to 6% in 2010.^23^ Comparison between termination rates reported prior to the introduction of NIPT with termination rates seen in the reviewed studies and audits suggests a general trend towards a decrease in termination rates compared with pre‐NIPT rates (Table 2). Data from the UK audit and research studies^35^, ^36^, ^37^ indicate that termination rates for women having NIPT are considerably lower than the pre‐NIPT termination rate of 92%.^21^ For the US^8^, ^28^, ^29^, ^31^, ^33^ and Dutch^34^ studies, decreases in the termination rate compared to pre‐NIPT rates were also seen. Notably in the United States, termination rates following a prenatal diagnosis of DS are already low (67%) compared to other countries (>90%). In some countries termination rates were unchanged, for example, in Hong Kong, in the studies reported by Lau et al,^26^, ^27^ a termination rate of 100% is in line with reported termination rates above 90% for mainland China.^24^

**Table 2:** Termination rates determined from reviewed studies and the audits reported here compared to pre‐NIPT termination rates

Citation	Termination Rate^a^	Country	Pre‐NIPT Termination Rate for DS^b^ (year(s) obtained)
Lau et al^26^, ^27^	100%^c^	Hong Kong	Not found
Pettit et al^28^	63%	United States	67% (1995‐2011)^41^
Vahanian et al^29^	0%	United States	67% (1995‐2011)^41^
Shaw et al^30^	100%^c^	Taiwan	68% (2001)^45^
Beamon et al^31^	60%	United States	67% (1995‐2011)^41^
Tiller et al^8^	40%	United States	67% (1995‐2011)^41^
Song et al^32^	67%	China	94% (2003‐2011)^24^
Dobson et al^33^	Singletons: 64% Twins: 40%	United States	67% (1995‐2011)^41^
Oepkes et al^34^	86%^c^	The Netherlands	93% (2010)^44^
Chitty et al^35^	NIPT: 70% IPD: 93% Overall: 79%	United Kingdom	92% (2007‐2008)^21^
Gil et al^36^, ^37^	NIPT:44% IPD: 93% Overall: 73%	United Kingdom	92% (2007‐2008)^21^
Gil et al^38^	100%^c^	Spain	96%^d^ (2002‐2004)^46^
Qiang et al^40^	87%	China	94% (2003‐2011)^24^
Le Conte et al^39^	100%^c^	France	96%^e^ (2002‐2004)^46^
Reported here	NIPT: 67% IPD: 93% Overall: 88%	Singapore	Not found
Reported here	62.7%	United Kingdom	92% (2007‐2008)^21^

a Termination rate calculated as a proportion of all pregnancies that had a high‐risk result for DS from NIPT (false positives and negatives excluded).

b Termination rate calculated as a proportion of all pregnancies that had a definitive prenatal diagnosis of DS by IPD.

c All high‐risk results from NIPT were confirmed by IPD.

d Termination rate for EUROCAT (European Surveillance of Congenital Anomalies) registry regions of Spain (Barcelona, Basque, Asturias, and Madrid).

e Termination rate for EUROCAT (European Surveillance of Congenital Anomalies) registry regions of France (Auvergne, Paris, Central East and Strasbourg).

Abbreviations: DS, Down syndrome; IPD, invasive prenatal diagnosis; NIPT, noninvasive prenatal testing.

It is important to be cautious when interpreting comparisons between the studies discussed here and historical controls, as numbers are small in the included studies and there may be differences between the populations used for determining the historical termination rates and those of the included studies, for example, in the systematic review of termination rates from the United States, Natoli et al^41^ noted that the summary termination rate that the authors calculated may not be applicable to the entire US population. In addition, the termination rates from the historical controls only include women with a confirmed prenatal diagnosis of DS. To get a complete picture of the impact of NIPT we have calculated termination rates for all women with a high‐risk NIPT result, not all of whom chose to have confirmatory invasive testing. For some studies included in the review there are no differences in how termination rates were calculated compared to historical controls, as all women in the study had their high‐risk NIPT result confirmed.^26^, ^27^, ^30^, ^34^, ^38^, ^39^ To look at termination rates across subgroups in the remaining studies, we could calculate the termination rates for women with and without a confirmed prenatal diagnosis of DS separately. However, this is not possible for all studies as the required information was either not reported or participant numbers were too low to allow a meaningful breakdown. In Chitty et al^35^ the termination rates for women with and without a confirmed diagnosis after a high‐risk NIPT result were 83% (29/35) and 12.5% (1/8), respectively, and in the UK audit 88% (21/24) and 8% (1/13), respectively. In the US study reported by Dobson et al,^33^ the termination rates for women with and without a confirmed diagnosis were 80% (28/35) and 27% (4/15), respectively. Notably, Dobson et al^33^ concluded, as we did, that the overall termination rate was not higher than historical controls and stated that their findings argue against the concern that cfDNA screening would increase rates of pregnancy termination.

As one of the common reasons women have declined screening in the past is the miscarriage risk associated with invasive testing,^47^, ^48^ it is not surprising that NIPT is being adopted widely around the world.^1^ However, our findings suggest that this increase may not impact greatly on the number of babies born with DS as many parents will use NIPT for information and not for decisions about termination of pregnancy. The decrease in termination rates compared with pre‐NIPT rates observed in the reviewed studies, presumably reflects, at least in part, the uptake of NIPT by women seeking information who would not have had prenatal testing in the past as they would not put their pregnancies at risk with invasive testing. We also found evidence that the termination rate for women opting for NIPT was lower when compared to women who chose to go directly to invasive testing following a high‐risk screening result. In the Singapore audit the number of women opting for termination following NIPT (4/6—66.7%) was lower than those who chose invasive testing (25/27—92.6%). Similarly, in the United Kingdom, in 2 prospective studies where NIPT was offered to both high and intermediate risk women the number of live births of children with DS were significantly higher amongst women opting for NIPT compared to women who chose to go straight to invasive testing.^35^, ^36^, ^37^ This difference is most likely due to the variances in motivation for women choosing NIPT versus invasive testing, with the latter group perhaps being more likely to want diagnostic information to make decisions about termination.^49^

We know from the literature that the uptake of NIPT is high,^34^, ^35^ making it likely that detection of DS will increase, but the lower termination rates following NIPT in some countries suggest that live birth rates may remain largely unchanged compared to termination rates prior to the introduction of NIPT. Termination rates did not, however, fall in all studies reviewed here and in settings where NIPT uptake is high and termination rates remain unchanged there will be an overall increase in numbers of terminations of pregnancy and a corresponding decrease in the live birth rate.

Ultimately, however, the research included here describes relatively small numbers of women and can only give insights into pregnancy outcomes following prenatal testing and we do not know how many children with DS were born to parents who chose not to have prenatal testing. Population‐based studies of live birth rates are therefore essential to allow us to see the overall impact of NIPT. The importance of the population‐based studies is highlighted by reports from countries such as the Netherlands where the termination rate following a prenatal diagnosis of DS is high, but the overall live birth rate has increased over time as many women opt not to have DS screening.^20^ Uptake of DS screening is low in the Netherlands compared to other European countries. This may reflect the fact that parents must make a financial contribution to screening, also that the offer of DS screening is not presented as a routine test and is discussed in a way that emphasises the right not to know.^50^ Moreover, several studies looking at hypothetical choices have shown that even with NIPT as an option many women will still choose not to have any prenatal testing. In a survey of 2666 women from 9 countries, there was a sizable proportion of women who said they would not have any prenatal testing for DS, including more than one third of women in the Netherlands and Israel.^51^ Similarly, studies from the United States and the Netherlands found that around one third of people surveyed reported not wanting any tests for DS.^52^, ^53^

Research looking at women's hypothetical choices regarding how they would respond to an NIPT result that suggests DS is highly likely support our findings that many women will choose to continue their pregnancies.^18^, ^49^, ^52^, ^54^ While the data on hypothetical choices needs to be interpreted with caution, as parents may make different choices when faced with real‐life situations, these studies indicate that a significant number of parents would use NIPT for information only, so that they could plan and prepare for the birth of an affected child rather than using the information to make decisions about termination of pregnancy.^18^, ^52^, ^55^, ^56^ The anticipated high uptake of NIPT suggested in these studies indicates that parents value having screening tests available, regardless of their intention to either terminate or continue an affected pregnancy. Recent research in the United States that surveyed 217 individuals, representative the US population by gender, income, and education, found that the majority (65%) saw the value of having reliable information when making health care decisions and therefore supported having NIPT and other prenatal tests available.^57^

Decision making about next steps following a diagnosis of DS is complex. Every woman must have access to the support they need to make informed decisions that take into consideration their own circumstances, experiences, the needs of their family and are in keeping with their personal beliefs and values.^58^, ^59^ Recent research has highlighted best care practices following a prenatal diagnosis of aneuploidy that include the provision of clear, accurate, and respectful communication about the testing process and results; empathic, nonjudgemental professional support; timely access to services; health professional acknowledgement of the enormity of the decision; and opportunities to discuss the diverse range of feelings that accompany prenatal diagnosis.^59^ As many parents will have prenatal testing with NIPT for information only, there will be more parents continuing pregnancies knowing that the baby has DS. As many of these women receive the diagnosis in early pregnancy it is important that their ongoing needs for emotional and clinical support are met. Furthermore, knowledge of fetal DS status may allow increased surveillance to prevent intrauterine death as highlighted by recent research from the United States, which found elevated rates of growth restriction, early delivery due to nonreassuring fetal status, and placental insufficiency in a cohort of 64 women continuing the pregnancy with a diagnosis of DS.^60^ Provision of practical and psychosocial support for individuals with DS and their families will also continue to be needed.

## 5 CONCLUSIONS

Parents value having the option of NIPT so that they can obtain information about DS early in pregnancy without putting their pregnancy at risk of miscarriage. The data presented here suggest that parents choose NIPT for different reasons, and a significant number of parents will use the results for information so that they can prepare for the birth of a child with DS. Comparison of termination rates reported in the studies reviewed here with termination rates reported prior to the introduction of NIPT suggest that in many settings the implementation of NIPT may not alter the overall numbers of children born with DS. Long‐term population‐based studies are needed to accurately determine the impact of NIPT on the number of children born with DS. Future research should also consider the implications for cost‐effectiveness and service provision of NIPT being used for information and planning as well as to direct decisions about termination of pregnancy.

## CONFLICTS OF INTEREST

J.F. is used by the charity Antenatal Results and Choices (ARC), which in the past has received small amounts of funding from some commercial companies marketing NIPT. All other authors declare no conflict of interest.
